# Enhancement of fatigue resistance by recrystallization and grain growth to eliminate bonding interfaces in Cu–Cu joints

**DOI:** 10.1038/s41598-022-16957-y

**Published:** 2022-07-30

**Authors:** Jia-Juen Ong, Dinh-Phuc Tran, Man-Chi Lan, Kai-Cheng Shie, Po-Ning Hsu, Nien‑Ti Tsou, Chih Chen

**Affiliations:** 1 Department of Materials Science and Engineering, National Yang Ming Chiao Tung University, Hsinchu, 30010 Taiwan; 2 Department of Materials Science and Engineering, National Chiao Tung University, Hsinchu, 30010 Taiwan

**Keywords:** Electrical and electronic engineering, Materials for devices, Structural materials

## Abstract

Cu–Cu joints have been adopted for ultra-high density of packaging for high-end devices. However, cracks may form and propagate along the bonding interfaces during fatigue tests. In this study, Cu–Cu joints were fabricated at 300 °C by bonding 〈111〉-oriented nanotwinned Cu microbumps with 30 μm in diameter. After temperature cycling tests (TCTs) for 1000 cycles, cracks were observed to propagate along the original bonding interface. However, with additional 300 °C-1 h annealing, recrystallization and grain growth took place in the joints and thus the bonding interfaces were eliminated. The fatigue resistance of the Cu–Cu joints is enhanced significantly. Failure analysis shows that cracks propagation was retarded in the Cu joints without the original bonding interface, and the electrical resistance of the joints did not increase even after 1000 cycles of TCT. Finite element analysis was carried to simulate the stress distribution during the TCTs. The results can be correlated to the failure mechanism observed by experimental failure analysis.

## Introduction

Moore’s law is widely considered as a predicting tool to observe the increase of transistor counts in a constant area since the first logic chip was invented^1^. They would double about every two years. To gain the performance of chips, three-dimensional (3D) integration is one of the most practical solutions^2–6^. In the semiconductor industry, transient liquid phase (TLP) bonding using different composition solders is a conventionally reliable technique showing low processing cost^7–9^. However, as shrinking the size of solder bumps to below 20 μm, the reliability issues related to intermetallic compounds (IMCs)^10,11^ and whiskers^12^, thermal fatigue^13–15^, sidewall wetting effects^16^ and Kirkendall voids are of concerns^17^. Such issues have restricted the shrinking efforts. Currently, solid-state bonding of metal/metal or metal/dielectric materials (hybrid bonding) is demonstrated as a crucial technology for advanced ultra-fine-pitch packaging.

Copper (Cu) with great electrical and thermal conductivity, electromigration resistance^18–21^ and mechanical strength is widely employed in metal direct bonding^22^ or hybrid bonding^23–25^. Currently, Cu/SiO_2_ hybrid bonding is already in the production line of complementary metal-oxide semiconductor (CMOS) image sensors by SONY Inc.^26^. The physical properties of fine-pitch Cu/SiO_2_ hybrid bonding with low electrical contact resistivity have been reported recently^27^. The bonding temperature of those joints is still in need of consideration^23^. Previously, nanotwinned Cu (nt-Cu) with highly 〈111〉-preferred^28^ orientation was found to have the highest surface diffusivity^29^ and lowest oxidation rate^30^ compared to coarse-grained Cu. Their electrical properties are also comparable^31^. Thus, it is expected for use in low temperature bonding for the next generation packaging^32^.

In this study, we tailored the microstructures of Cu microbumps with a diameter of 30 μm by proper thermal annealing, and applied stress to the Cu microbumps through temperature cycling tests. Failure analyses were then executed using scanning electron microscope (SEM), focused ion beam (FIB), electron backscattered diffraction (EBSD) to correlate the reliability of the joints and failure mechanisms with the status of bonding interfaces. Finite element method (FEM) was also employed to analyze stresses in the joints during thermal cycling. The mechanisms of crack initiation and propagation were proposed.

## Experimental and numerical analysis

In this study, 〈111〉-oriented nt-Cu microbumps were electroplated onto an 8-inches photoresist (PR) patterned wafer of a silicon substrate using direct current (DC) at room temperature. The thickness of the Si substrate was 500 μm with 200-nm SiO_2_, 100-nm adhesion layer (Ti) and 200-nm Cu seed layer. A 99.99% Cu bulk was used as the electroplating anode. The electrolyte consisted of a high-purity CuSO_4_ solution with 0.8 M of Cu cations, 0.1 mL/L of hydrochloric acid (HCl). The use of HCl was to promote Cu crystallinity and deposition rate. An additive (108C, Chemleaders Corporation, Hsinchu, Taiwan) was also employed for nt-Cu nucleation.

**Figure 1:**
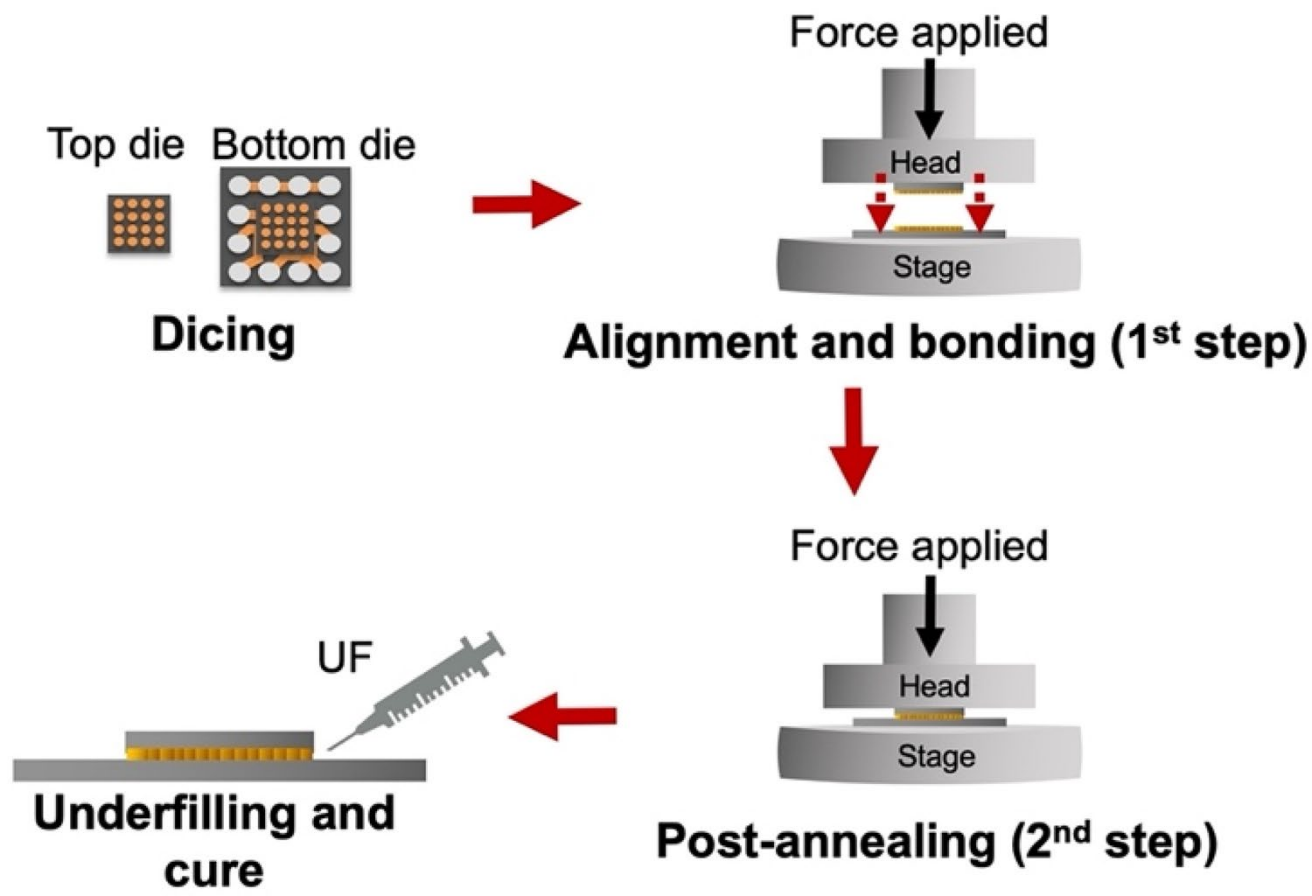
Fabrication flow of the two-step bonding procedure of nt-Cu microbumps.

Prior to the first-step bonding, chemical mechanical planarization (CMP) was applied to planarize the surface of the bumps. Figure 1 illustrates the fabrication flow of the joints. The wafer was diced into sizes of 6 × 6 mm^2^ and 15 × 15 mm^2^ for the top and bottom dies, respectively. We used citric acid^33,34^ to clean residual Cu oxides. Such oxides may act as a diffusion barrier and thus reduce the bonding quality. The cleaned dies were then placed on the stage of a manual alignment bonding machine (CA-2000VA, Bondtech Co., Japan) for thermal compression bonding (TCB). We conducted the bonding by two steps. The parameters used were tabulated in Table 1. Then all the samples were post-annealed at 300 °C under 47 MPa for 1 h to enhance the bonding and tailor the interfacial microstructures. The gaps between the microbumps and Si substrates were then filled with underfill (UF, Eccobond UF 3915, Loctite Co., Germany) to bar oxidation. Subsequently, the UF and SiO_2_ particles were cured at 130 °C for 20 min.

**Table 1:** Bonding conditions in the 1st step process.

No.	Temperature (°C)	Bonding pressure (MPa)	Time (s)	Atmosphere
1	300	93	10	N_2_
2	300	47	10	N_2_
3	300	31	10	N_2_

Temperature cycling tests (TCTs) were then conducted on the microbumps to characterize and correlate the lifetime, thermal fatigue, and electrical performance. The temperature range of the TCTs was set from − 55 to 125 °C with a ramp rate of 15 °C/ min following a 5-min soaking for 1000 cycles. The electrical resistance was measured every 250 cycles using a 4-point probe method. Focused ion beam (FIB), scanning electron microscope (SEM), and electron backscattered diffraction (EBSD) were employed for microstructural and failure characterizations. The surface roughness of the Cu joints was analyzed by an atomic force microscope (AFM). Failure modes, fatigue behaviors, and crack formations during thermal cycling were then correlated with the changes of electrical resistance. Additionally, 3D FEM models were established for deeper understanding on stress distribution and failure mechanisms of the bumps during TCTs.

## Results and discussion

**Figure 2:**
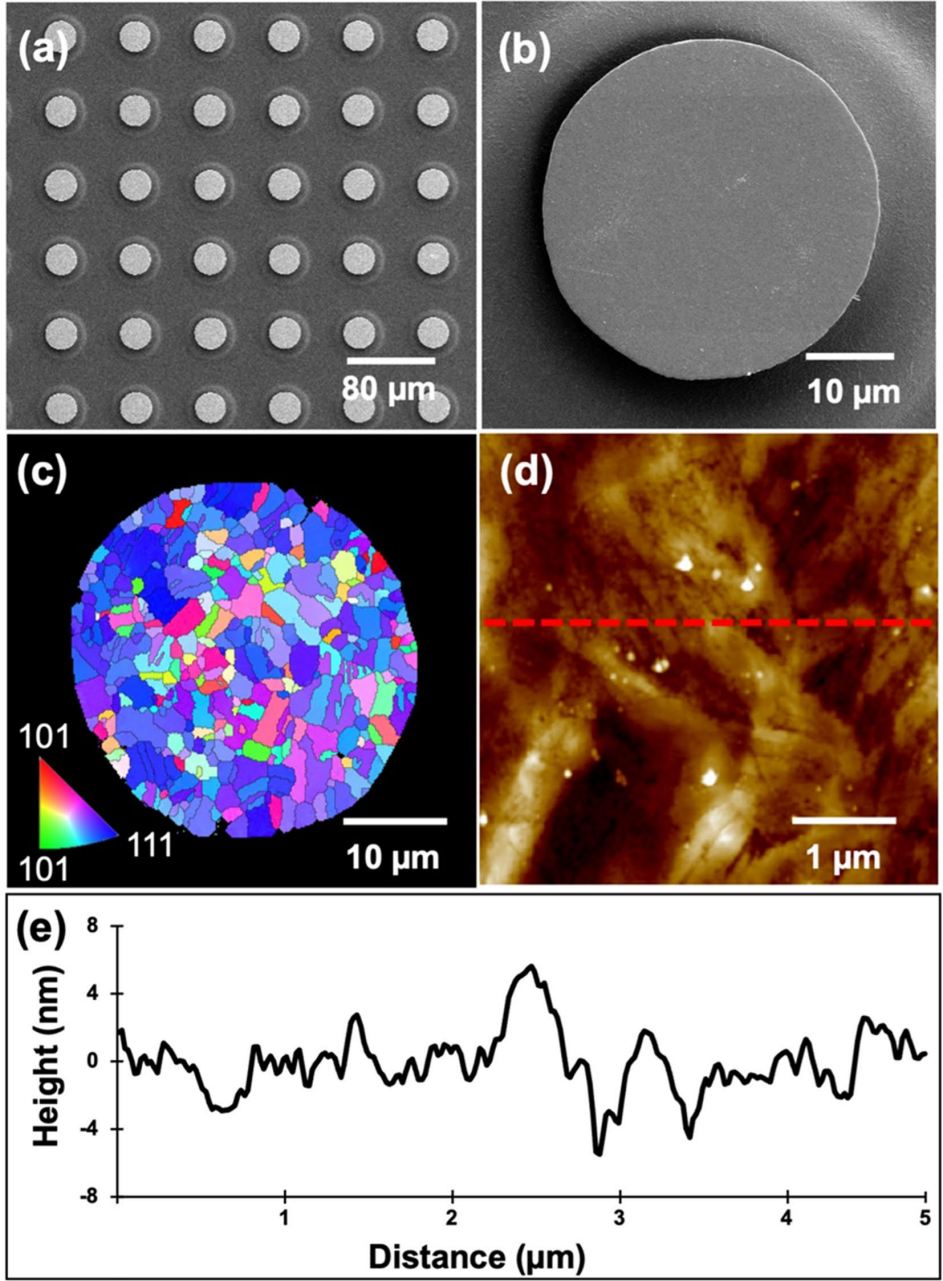
Top-view SEM micrograph of the (**a**) nt-Cu bump array with an enlarged image of a (**b**) single microbump flatted by CMP. (**c**) EBSD image at the normal direction (ND) of the bonding surface showing ~ 50% 〈111〉 orientation. (**d**) AFM micrograph taken in an area of 5 × 5 μm. The root mean square roughness (*R*_q_) of the bumps was ~ 4 nm. (**e**) Typical surface geometry of the microbumps scanned from the red dotted line in (**d**).

**Figure 3:**
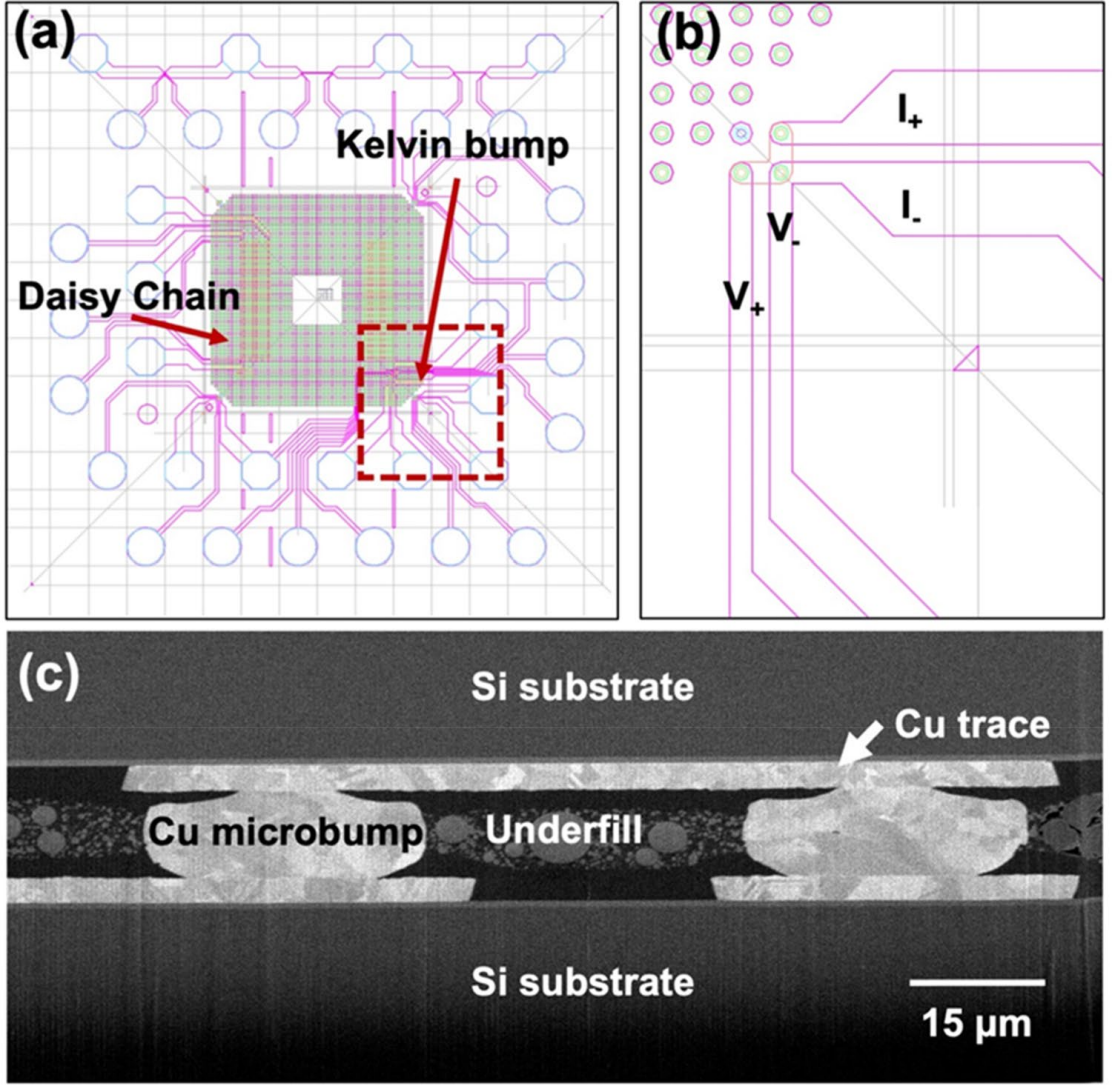
(**a**) Typical layout and (**b**) enlargement of the Kelvin microbump structure. (**c**) Cross-sectional SEM image of the Kelvin microbumps after 1000 thermal cycles.

**Figure 4:**
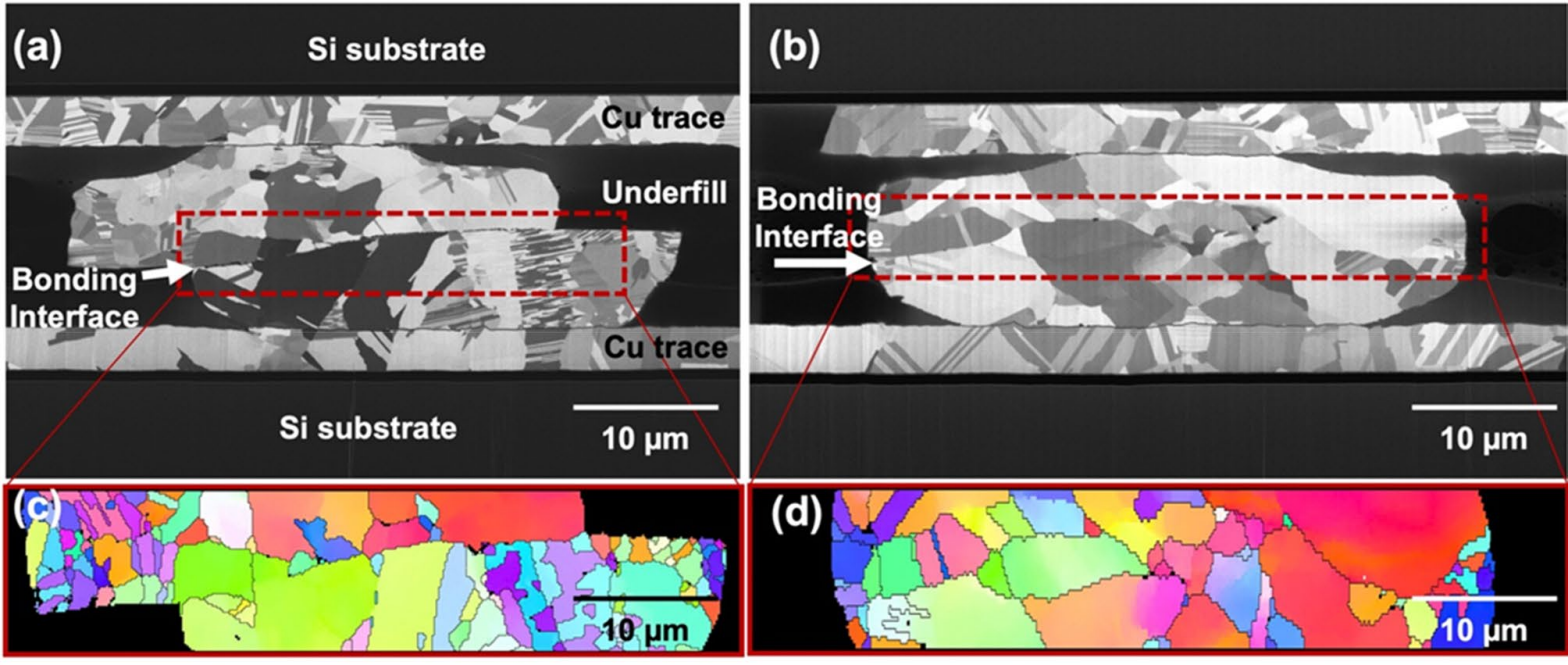
Ion FIB and EBSD images of the Kelvin microbumps bonded at 300 °C/47 MPa/10 s after 1000 thermal cycles: (**a**, **c**) as-fabricated and (**b**, **d**) post-annealed, respectively. It is obvious in (**c**) that cracks propagated along the bonding interface. As the original bonding interface was eliminated, no obvious cracks were detected (**d**). Thus, its electrical resistance was mostly maintained unchanged under thermal cycling.

It has been reported that 〈111〉-oriented nt-Cu possesses the highest surface diffusivity and lowest oxidation rate due to the highest packing density and lowest dangling bonds of the 〈111〉 surface^29,30,35^. Thus, bonding temperature or process time can be reduced using such nt-Cu films. Figure 2a and b shows the typical plan-view SEM images of the nt-Cu microbumps. The microbump thickness was ~ 7 µm. Approximate 50% of 〈111〉 orientation was observed by orientation imaging microscopy (OIM), as illustrated in Fig. 2c. Note that the roughness of the joints also plays a key role on bonding quality and oxidation resistance^36,37^. Thus, we planarized the Cu joints using CMP. As shown in Fig. 2d–e, a small-scale root mean square roughness (*R*_q_) of ~ 4 nm was achieved. The layouts of the test vehicles and electrical structure with a typically cross-sectional SEM image of the microbumps are shown in Fig. 3. The test vehicles consisted of four Kelvin bumps and daisy chains (40 and 400 bumps). The diameter and height of the nt-Cu microbumps were ~ 30 and 7 μm, respectively. These test vehicles were used to investigate the changes in electrical resistance of such microbumps under thermal cycling. In this study, we heat-treated the Cu microbumps to eliminate the bonding interface. As shown in Fig. 4, the bonding interface still existed in the as-fabricated bumps (Fig. 4a,c), while that in the post-annealed ones was eliminated (Fig. 4b,d). Such interfacial elimination resulted from the recrystallization and grain growth in the bumps. These microstructural changes apparently enhanced the bonding strength of the Cu–Cu joints^38^.

**Figure 5:**
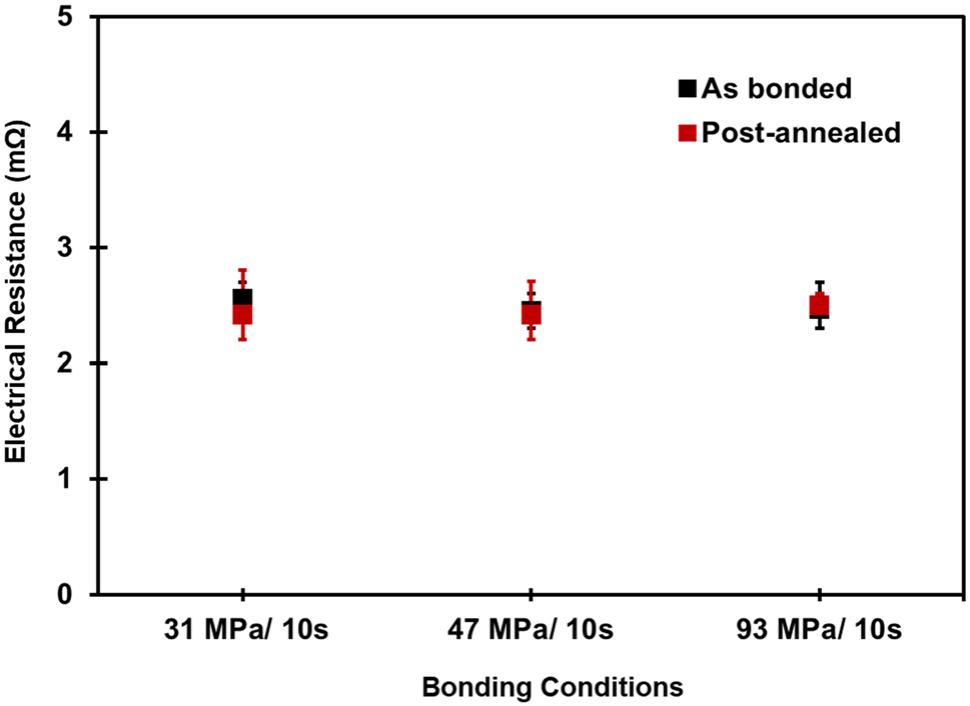
Variation of the electrical resistance of the as-fabricated and post-annealed joints bonded at different bonding conditions. These values are comparable.

**Figure 6:**
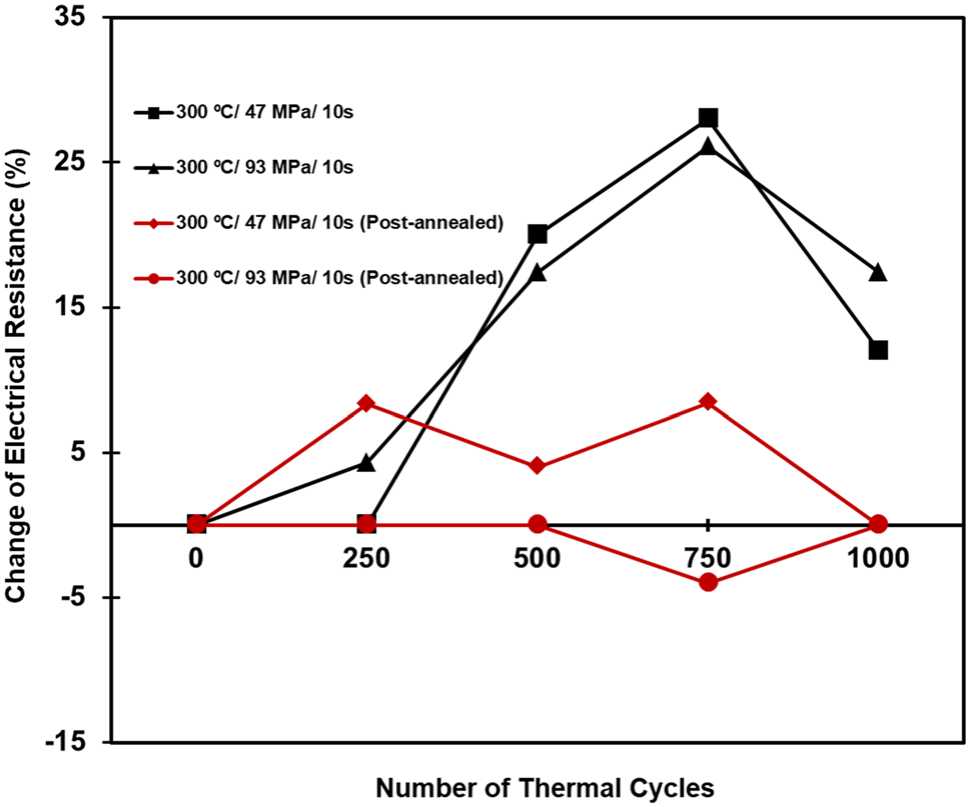
Changes in electrical resistance of the joints bonded at different conditions. The electrical resistance of the post-annealed joints remained almost unchanged after 1000 thermal cycles.

We also performed TCTs to further study the fatigue behaviors, crack initiation and propagation, and to correlate with their electrical resistances. As shown in Fig. 5, the electrical resistances of the as-fabricated and post-annealing joints were comparable. Similar phenomenon has been also reported by Nitta et al.^39^. They found that, above 12 K, the electron scattering by thermal lattice vibration was the primary factor used to determine electrical resistance. Although the total area of grain boundaries (GBs) decreased and the original interface was eliminated by triggering the recrystallization and interfacial grain growth, it was still hard to detect any difference in resistivity at room temperature. When subjecting the joints to thermal cycling, differences in the electrical resistance changes became more obvious. The changes of electrical resistance of the joints with and without post-annealing under thermal cycling are shown in Fig. 6. It can be seen that the electrical resistance of the post-annealed joints after 1000 thermal cycles maintained approximately unchanged while that of the joints without post-annealing significantly increased (to 12% and 17.4% as bonded at 47 and 93 MPa, respectively). Obviously, the post-annealing could enhance the resistance to thermal fatigue.

**Figure 7:**
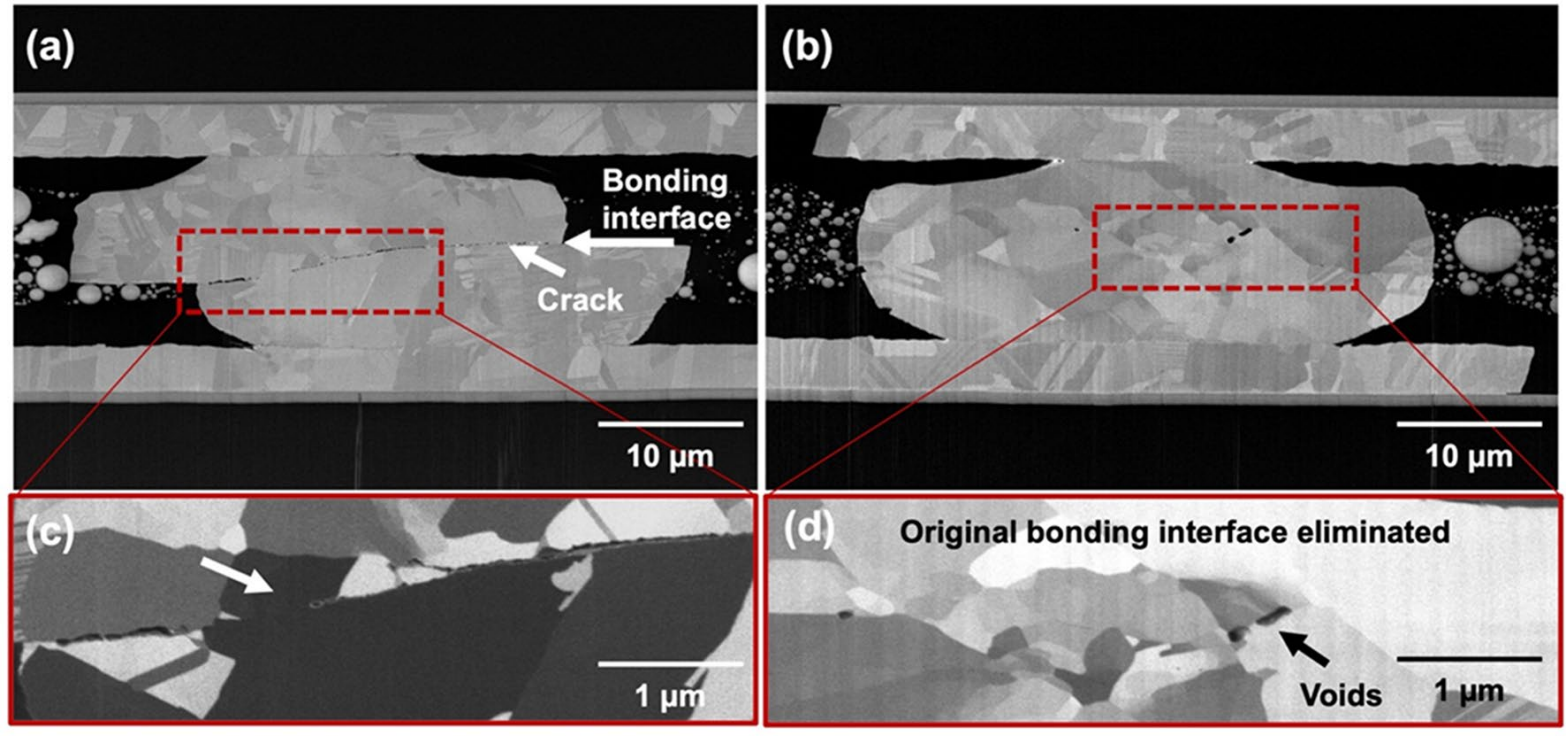
Cross-sectional FIB micrographs of the Cu joints bonded at 300 °C/47 MPa/10 s after 1000 thermal cycles: (**a**, **c**) the as-fabricated and (**b**, **d**) post-annealed samples. (**a**, **b**) The white arrows denote the original bonding interfaces. (**c**) The enlarged ion image of the (**a**) as-fabricated joints. Only one recrystallized grain grew across the interface. Cracks initiated and propagated along the bonding interface. (**d**) The enlarged ion image of the (**b**) post-annealed Cu joint. Only a few voids at the grain boundaries without cracks were observed in the post-annealed joints.

In order to further characterize the effect of post-annealing on the fatigue behaviors of the joints, FIB analysis was conducted. The cross-sectional FIB images of the Cu joints bonded at 300 °C/47 MPa/10 s with and without post-annealing after 1000 thermal cycles are shown in Fig. 7. In the 1st-step annealed samples (Fig. 7a,c), a few of nanotwinned columnar grains recrystallized and grew across the bonding interface. Crack propagation ceased in such areas. In the post-annealed samples (Fig. 7b,d), a large amount of columnar grains was consumed, recrystallized and grew across the bonding interface. The annealing time and applied pressure were sufficient causing atomic diffusion at the bonding interface^40,41^. The original bonding interface was eliminated and replaced by newly recrystallized grains. It prevented crack formation and propagation in the joints under the thermal cycling.

**Figure 8:**
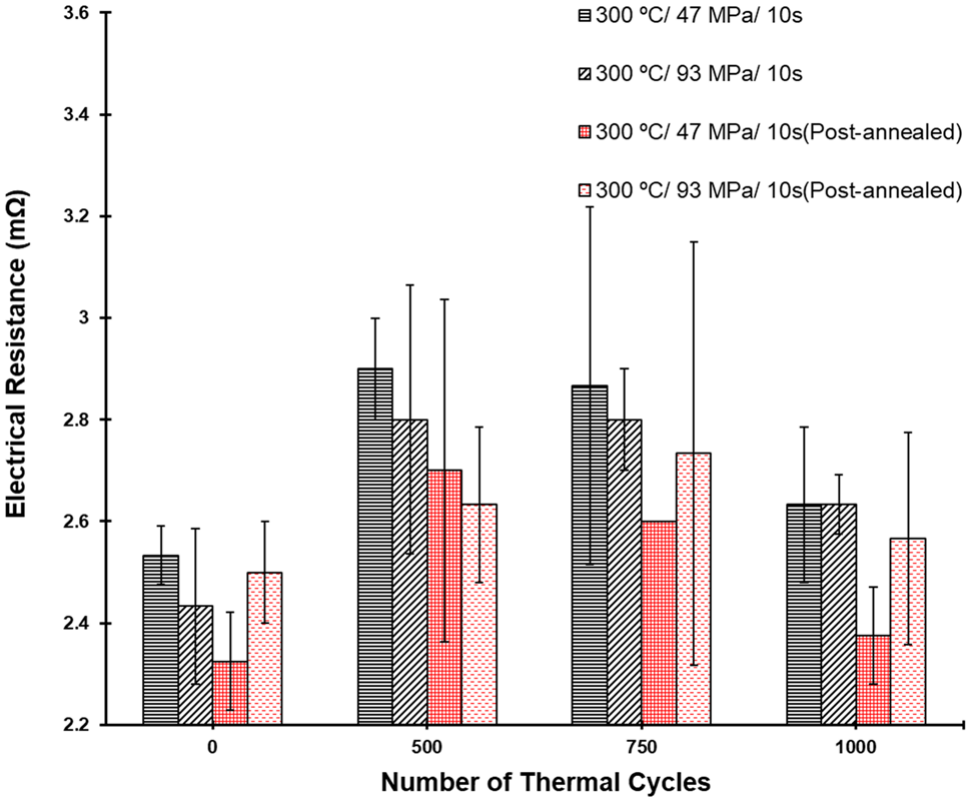
Electrical resistance of the Cu joints bonded at various conditions. They were measured after some specific numbers of thermal cycles.

To examine the fatigue lifetime of the joints, we measured their electrical resistances after some specific numbers of thermal cycles and plotted in Fig. 8. As aforementioned, the differences of the electrical resistance among the as-bonded samples measured by a 4-point probe method were not obvious. However, obvious differences were seen after 500 thermal cycles or more. Apparently, the electrical resistance of the post-annealed joints was lower than that of the as-fabricated counterparts. Under thermal fatigue, cracks in the interfaces of the joints without post-annealing tended to initiate and propagate resulting in the obvious increase in electrical resistance after 500 cycles (Fig. 8). Interestingly, the electrical resistance of the post-annealed microbumps did not clearly increase after 1000 thermal cycles. As aforementioned, their bonding interfaces were eliminated by the post-annealing, and crack initiation and propagation could be suppressed. This explains why the post-annealed microbumps were more resistant to thermal fatigue than the as-fabricated counterparts. We found that the electrical resistance of the joints generally dropped after 750 thermal cycles. This could be attributed to the recovery behaviors^42^ of such a metal and the effect of mechanical annealing^43,44^. During the recovery process, the stored internal strain energy is relieved by dislocation motions (without an externally applied loading). This is further facilitated by atomic diffusion at an elevated temperature. Additionally, defects (dislocations) intrinsically existed in the metal during fabrication processes. Under an applied thermal stress (the mechanical stress induced by the changes in temperature), such a metal tends to lower down its defect density. In this study, under the thermal cycling (− 55 to 125 °C), thermal stress gradients also induced in the Cu joints. Thus, their physical properties (electrical and thermal conductivities) were recovered to the pre-cold-worked states. As shown in Figs. 6 and 7, the drops in the electrical resistance could be attributed to the aforementioned behaviors of the Cu joints.

**Figure 9:**
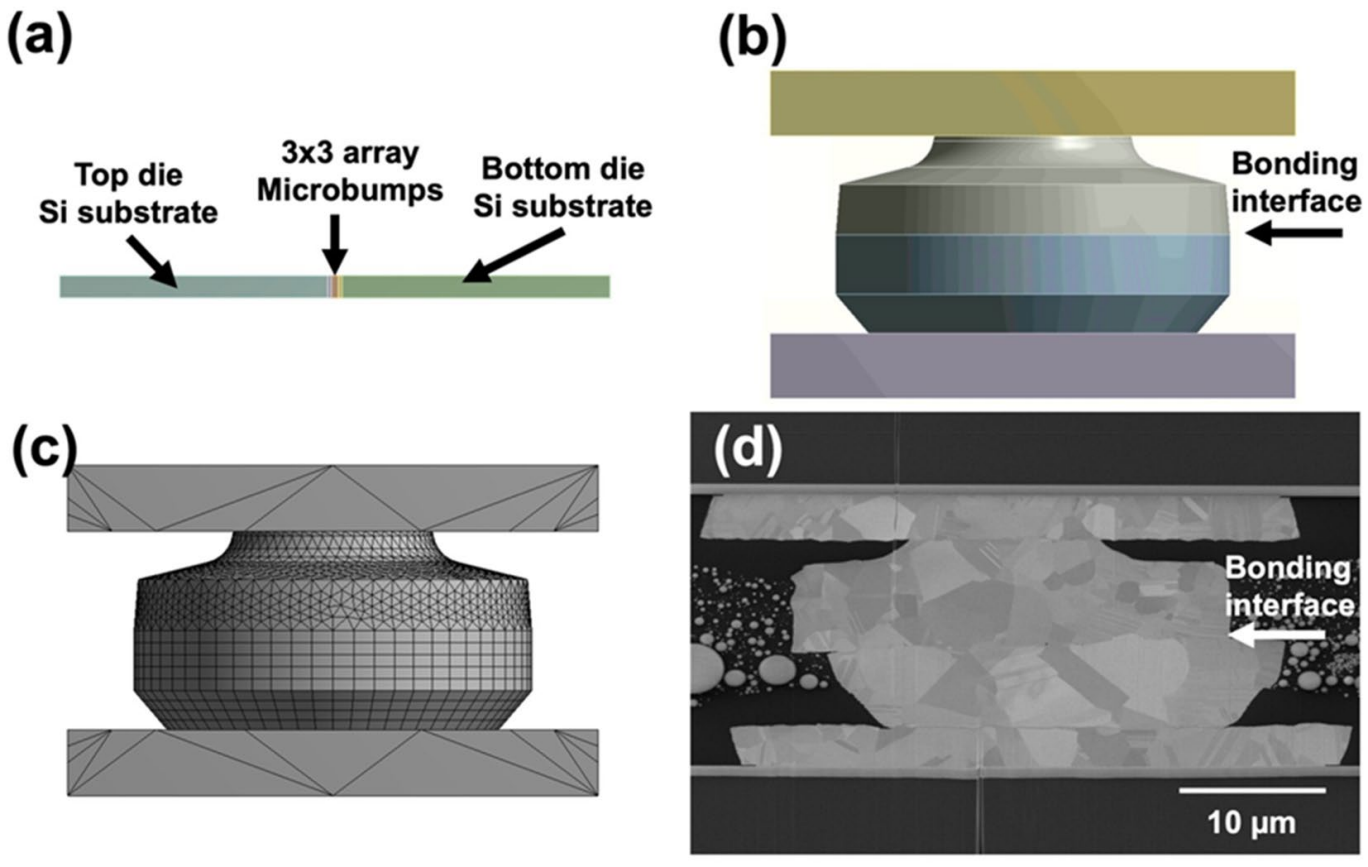
(**a**) 3D FEA model with 500-μm thick Si substrates at both sides. Symmetry was taken into account. (**b**) Cross-sectional view of the constructed 3D model of a single microbump. (**c**) Cross-sectional view of the model with meshing. (**d**) SEM image of the joints.

**Figure 10:**
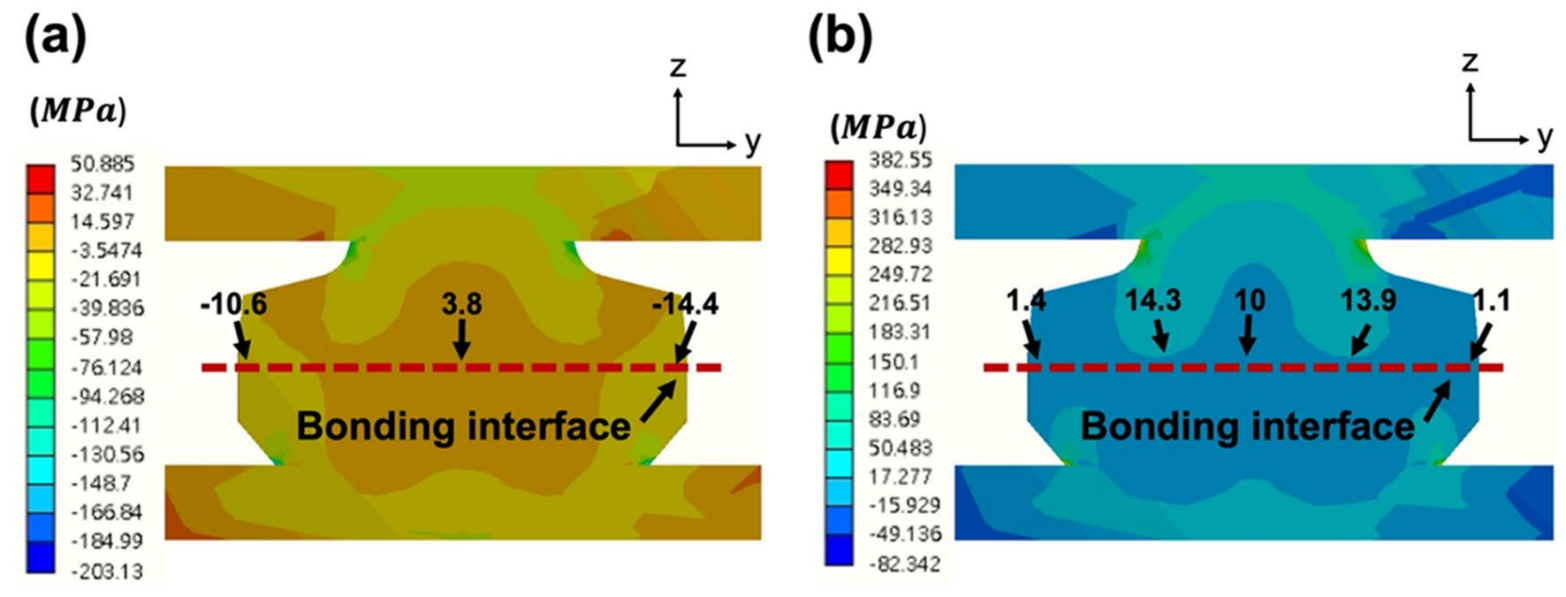
Stress distributions in the 3D FEA model at (**a**) − 55 °C and (**b**) 125 °C. The red dotted lines illustrate the bonding interfaces. The maximum and minimum stresses in the bonding interfaces are indicated by the black arrows.

To deeper understand the stress distribution induced during TCT, finite element analysis (FEA) was carried out. The material properties adopted in the simulation were listed in Table 2. Figure 9 shows the details of the FEA model resembling the representative microbump in Fig. 9d. The stress distributions in the model at – 55 and 125 °C are shown in Fig. 10. Under thermal cycling, stress is induced due to the large mismatch of the coefficient of thermal expansion (CTE) of Cu and UF^14,45,46^. At − 55 °C, a maximum tensile stress (3.8 MPa) formed at the center of the bonding interface (Fig. 10a). At 125 °C, it increased to 14.3 MPa (Fig. 10b). Such significant changes in stress at the bonding interface thus led to void and crack formations and finally caused failures of the joints. Note that the bonding interface is considered as the weakest location of the joints. Cracks tended to propagate along the straight bonding interface. In this study, we eliminated the bonding interface and recrystallized the grain boundaries by the 2-step bonding process (post-annealing). Under thermal fatigue, cracks would propagate along the recrystallized grain boundaries instead of the straight interface. The cracking paths would be extended. Thus, the post-annealed joints were more resistant to crack propagation resulting in their great reliability performance.

**Table 2:** Material properties employed in the FEM analysis.

Materials	*T*_g_ (℃)	CTE (ppm/℃)	Resistivity
Cu	–	16.8	1.7
PBO	300	64	–
Underfill	125	25 (< *T*_g_) 100 (> *T*_g_)	–

## Conclusion

In summary, Cu–Cu microbumps were fabricated using 〈111〉-oriented nt-Cu with 30 μm in diameter. Some microbumps were further annealed to trigger recrystallization and grain growth and to remove their bonding interfaces. The mechanisms of crack initiation and propagation caused by the CTE mismatch between Cu and UF during TCTs were then proposed. FEA was also performed to analyze the stress distribution in the joints under thermal cycling. The numerical results were then correlated with the failure mechanisms found in the experiments. We observed that cracks initiated and propagated along the continuous bonding interface of the joints without post-annealing. The recrystallization and grain growth in the post-annealed samples were favorable in barring crack formation and extensions. Given that the bonding interface was eliminated, cracks would propagate along zig-zag grain boundaries. They took longer pathways to further expand resulting in a greater resistance to crack propagation. Thus, the joints with a proper heat-treatment were more reliable under thermal fatigue than the as-fabricated counterparts.

## Data Availability

The raw/processed data required to reproduce these findings cannot be shared at this time as the data also forms part of an ongoing study.

## References

[1] Moore GE (1965). Cramming More Components onto Integrated Circuits.

[2] Zhang L (2018). Materials, processing and reliability of low temperature bonding in 3D chip stacking. J. Alloys Compd..

[3] Kurino, H. *et al.* in *International Electron Devices Meeting 1999. Technical Digest (Cat. No. 99CH36318)*, 879–882. 10.1109/IEDM.1999.824289 (2021).

[4] Ramm P (1997). Three dimensional metallization for vertically integrated circuits: Invited lecture. Microelectron. Eng..

[5] Iyer SS (2015). Three-dimensional integration: An industry perspective. MRS Bull..

[6] Chen C, Yu D, Chen K-N (2015). Vertical interconnects of microbumps in 3D integration. MRS Bull..

[7] Zeng K, Tu K-N (2002). Six cases of reliability study of Pb-free solder joints in electronic packaging technology. Mater. Sci. Eng. R Rep..

[8] Li J, Agyakwa P, Johnson C (2011). Interfacial reaction in Cu/Sn/Cu system during the transient liquid phase soldering process. Acta Mater..

[9] Li J, Agyakwa P, Johnson C (2010). Kinetics of Ag3Sn growth in Ag–Sn–Ag system during transient liquid phase soldering process. Acta Mater..

[10] Pang JH, Low T, Xiong B, Luhua X, Neo C (2004). Thermal cycling aging effects on Sn–Ag–Cu solder joint microstructure, IMC and strength. Thin Solid Films.

[11] Ma X, Qian Y, Yoshida F (2002). Effect of La on the Cu–Sn intermetallic compound (IMC) growth and solder joint reliability. J. Alloys Compd..

[12] Chuang T-H (2006). Rapid whisker growth on the surface of Sn–3Ag–0.5 Cu–1.0 Ce solder joints. Scr. Mater..

[13] Liang YC (2013). Anisotropic grain growth and crack propagation in eutectic microstructure under cyclic temperature annealing in flip-chip SnPb composite solder joints. Scr. Mater..

[14] Schubert, A. *et al.* in *Electronic components and technology conference*, 603–610. 10.1109/ECTC.2003.1216343 (1999).

[15] Pang JH, Chong D, Low T (2001). Thermal cycling analysis of flip-chip solder joint reliability. IEEE Trans. Compon. Packag. Manuf. Technol..

[16] Liang Y, Chen C, Tu K-N (2012). Side wall wetting induced void formation due to small solder volume in microbumps of Ni/SnAg/Ni upon reflow. ECS Solid State Lett..

[17] Kawano, M. in *2021 5th IEEE Electron Devices Technology & Manufacturing Conference (EDTM)*, 1–3. 10.1109/EDTM50988.52021.9421033 (2015).

[18] Shen F-C (2021). Atomic-scale investigation of electromigration with different directions of electron flow into high-density nanotwinned copper through in situ HRTEM. Acta Mater..

[19] Shie KC, Hsu PN, Li YJ, Tran DP, Chen C (2021). Failure mechanisms of Cu-Cu bumps under thermal cycling. Materials.

[20] Tran D-P, Li H-H, Tseng I-H, Chen C (2021). Enhancement of electromigration lifetime of copper lines by eliminating nanoscale grains in highly <111>-oriented nanotwinned structures. J. Mater. Res. Technol..

[21] Lloyd J, Clement J (1995). Electromigration in copper conductors. Thin Solid Films.

[22] Panigrahy AK, Chen K-N (2018). Low temperature Cu–Cu bonding technology in three-dimensional integration: An extensive review. J. Electron. Packag..

[23] Liu H-C, Gusak AM, Tu KN, Chen C (2021). Interfacial void ripening in Cu Cu joints. Mater. Character..

[24] Ko C-T, Chen K-N (2012). Low temperature bonding technology for 3D integration. Microelectron. Reliab..

[25] Kuo Y-H, Tran D-P, Ong J-J, Tu K, Chen C (2022). Hybrid Cu-to-Cu bonding with nano-twinned Cu and non-conductive paste. J. Mater. Res. Technol..

[26] Tian, Y. Low-temperature Cu/SiO2 hybrid bonding using a novel two-step cooperative surface activation. *ICEPT 2021*. 10.1109/ICEPT52650.52021.9568007 (2021).

[27] Ong J-J (2022). Low-temperature Cu/SiO_2_ hybrid bonding with low contact resistance using (111)-oriented Cu surfaces. Materials.

[28] Hsiao H-Y (2012). Unidirectional growth of microbumps on (111)-oriented and nanotwinned copper. Science.

[29] Liu CM (2015). Low-temperature direct copper-to-copper bonding enabled by creep on (111) surfaces of nanotwinned Cu. Sci. Rep..

[30] Tseng CH, Tu KN, Chen C (2018). Comparison of oxidation in uni-directionally and randomly oriented Cu films for low temperature Cu-to-Cu direct bonding. Sci. Rep..

[31] Lu L, Shen Y, Chen X, Qian L, Lu K (2004). Ultrahigh strength and high electrical conductivity in copper. Science.

[32] Tu K-N, Chen C, Chen H-M (2021). Electronic Packaging Science and Technology.

[33] Fujino M, Akaike M, Matsuoka N, Suga T (2017). Reduction reaction analysis of nanoparticle copper oxide for copper direct bonding using formic acid. Jpn. J. Appl. Phys..

[34] Koyama S, Hagiwara N, Shohji I (2015). Cu/Cu direct bonding by metal salt generation bonding technique with organic acid and persistence of reformed layer. Jpn. J. Appl. Phys..

[35] Liu C-M (2014). Low-temperature direct copper-to-copper bonding enabled by creep on highly (1 1 1)-oriented Cu surfaces. Scr. Mater..

[36] Lin P-F, Tran D-P, Liu H-C, Li Y-Y, Chen C (2022). Interfacial characterization of low-temperature Cu-to-Cu direct bonding with chemical mechanical planarized nanotwinned Cu films. Materials.

[37] Kim SJ (2022). Flat-surface-assisted and self-regulated oxidation resistance of Cu (111). Nature.

[38] Juang JY, Lu CL, Li YJ, Tu KN, Chen C (2018). Correlation between the microstructures of bonding interfaces and the shear strength of Cu-to-Cu joints using (111)-oriented and nanotwinned Cu. Materials.

[39] Nitta T, Ohmi T, Otsuki M, Takewaki T, Shibata T (1992). Electrical properties of giant-grain copper thin films formed by a low kinetic energy particle process. J. Electrochem. Soc..

[40] Ong J-J, Tran D-P, Yang S-C, Shie K-C, Chen C (2021). Shearing characteristics of Cu-Cu joints fabricated by two-step process using highly <111>-oriented nanotwinned Cu. Metals.

[41] Juang JY, Lu CL, Li YJ, Tu KN, Chen C (2018). Correlation between the microstructures of bonding interfaces and the shear strength of Cu-to-Cu joints using (111)-oriented and nanotwinned Cu. Materials.

[42] Callister WD, Rethwisch DG (2018). Materials Science and Engineering: An Introduction.

[43] Shan ZW, Mishra RK, Syed Asif SA, Warren OL, Minor AM (2008). Mechanical annealing and source-limited deformation in submicrometre-diameter Ni crystals. Nat. Mater..

[44] Shen YA, Chang L, Chang SY, Chou YC, Tu KN, Chen C (2022). Nanotwin orientation on history-dependent stress decay in Cu nanopillar under constant strain. Nanotechnology.

[45] Zhang, L. W., Wang, J. C., Yu, Q. & Meng, Q. D. *Advanced Materials Research*, 530–534. 10.4028/www.scientific.net/AMR.4314-4316.4530.

[46] Chen L, Zhang Q, Wang G, Xie X, Cheng Z (2001). The effects of underfill and its material models on thermomechanical behaviors of a flip chip package. IEEE Trans. Adv. Packag..

